# Improving acne severity detection: a GAN framework with contour accentuation for image deblurring

**DOI:** 10.3389/fbinf.2025.1485797

**Published:** 2025-03-10

**Authors:** Philomina Princiya Mascarenhas, M. S. Sannidhan, Ancilla J. Pinto, Dabis Camero, Jason Elroy Martis

**Affiliations:** ^1^ Department of Computer Science and Engineering, NMAM Institute of Technology (Nitte Deemed to be University), Nitte, India; ^2^ Department of Computer Science and Engineering, Manipal Institute of Technology, Manipal Academy of Higher Education, Manipal, India; ^3^ Department of Electrical Engineering, Penn State University Harrisburg, Middletown, PA, United States; ^4^ Department of Information Science and Engineering, NMAM Institute of Technology (Nitte Deemed to be University), Nitte, India

**Keywords:** generative adversarial networks, image deblurring, contour accentuation technique, teledermatology, acne severity detection

## Abstract

Teledermatology, a growing field of telemedicine, is widely used to diagnose skin conditions like acne, especially in young adults. Accurate diagnosis depends on clear images, but blurring is a common issue in most images. In particular, for acne images, it obscures acne spots and facial contours, leading to inaccurate diagnosis. Traditional methods to address blurring fail to recover fine details, making them unsuitable for teledermatology. To resolve this issue, the study proposes a framework based on generative networks. It comprises three main steps: the Contour Accentuation Technique, which outlines facial features to create a blurred sketch; a deblurring module, which enhances the sketch’s clarity; and an image translator, which converts the refined sketch into a color photo that effectively highlights acne spots. Tested on Acne Recognition Dataset, the framework achieved an SSIM of 0.83, a PSNR of 22.35 dB, and an FID score of 10.77, demonstrating its ability to produce clear images for accurate acne diagnosis. Further, the details of research can be found on the project homepage at: https://github.com/Princiya1990/CATDeblurring.

## 1 Introduction

In digital image processing, clear images are essential for accurate analysis and decision-making in many areas (Bui et al., 2018). However, blurring caused by camera movement or focus problems can distort important details (Pan et al., 2019). This is especially problematic in teledermatology, where clear images are important for evaluating skin conditions, such as acne severity, to ensure accurate diagnoses, proper treatment, and avoid misunderstandings (Suha and Sanam, 2022). Considering this, there is a strong need for effective image processing methods to improve image clarity and retain fine details.

Straight forward approaches for deblurring, such as Wiener filtering and Richardson-Lucy deconvolution (Šroubek et al., 2019), rely on mathematical models to reverse blurring by estimating and correcting the distortion, aiming to minimize the mean square error between the restored and original images (Gnanasambandam et al., 2024). However, these methods struggle with complex blurring or unpredictable noise, often resulting in issues like ringing artifacts and amplified noise (Joshi and Sheetlani, 2019). These limitations underscore the need for more adaptive and reliable approaches, with studies highlighting GAN-based deblurring techniques as a promising solution to overcome these challenges by restoring image clarity (Mankotia et al., 2024; Song and Lam, 2021). However, some studies also revealed that GANs suffer to generate better quality of deblurred images due to the insufficient exposure of facial structure hiding the contours in the blurred images (Shi et al., 2024; Qi et al., 2021; Jung et al., 2022). This limitation is particularly significant in applications like acne assessments, where facial contours provide major context for understanding lesion distribution and texture (Samizadeh, 2022). One commonly used technique to address this issue is translating the image into a sketch, which helps to sharpen the image by emphasizing its contours. However, when applied to blurred images, this approach often fails to produce clear sketches due to the insufficient visibility of facial structures (Chen et al., 2024). To address these challenges, implementing an image processing algorithm or preprocessor that exposes contours before sketching can significantly improve the clarity of sketches. This approach ensures better visualization and more accurate results, particularly in applications like dermatological assessments, where clear contour details are essential for understanding conditions like acne (Dumitrescu et al., 2021).

**FIGURE 1:**
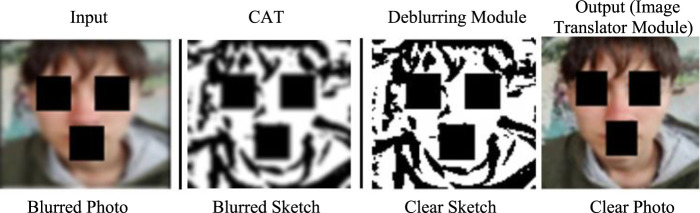
Sample image showing the workflow of the proposed image deblurring framework.

Building on the observations discussed earlier, the proposed image deblurring framework is designed to overcome the limitations of existing systems through three main components. The first is an image preprocessor called the Contour Accentuation Technique (CAT), which extracts the contours of facial structures from a blurred input image, presenting them in the form of a sketch. Next, a deblurring module featuring DeblurGAN refines the sketch produced by CAT, removing blur and enhancing clarity. Finally, an image translator module, powered by a conditional GAN (cGAN), converts the clear sketch into a color photo image. Although the CAT component initially generates a blurry sketch, it effectively highlights the facial contours, providing structural information that aids the deblurring module in producing a higher-quality sketch. These refined sketches, when translated into photo images by the image translator module, result in images where acne spots are clearly visible, improving their overall diagnostic utility. Figure 1 provides an overview of this process, showing the progression from deblurred input photo to clear photo image as output.

The effectiveness of the proposed image deblurring framework is evaluated using the Acne Recognition Dataset (Kucev, 2023) as the primary benchmark, specifically designed for acne severity detection. For further validation, standard datasets: CUHK (Wang and Tang, 2008), AR (Cao et al., 2022), and XM2GTS derived from a subset XM2VTS dataset (Messer et al., 1999) are used.

This research work has the following research contributions:

1. Implementation of CAT to reveal facial structures through sketch generation based on contour extraction.
2. Utilization of DeblurGAN to specifically enhance the clarity of blurred sketches generated by CAT in order to highlight acne spots.
3. Development of an image translator module using cGAN to transform sketches into color photo images, enabling a realistic visual analysis of acne spots.

## 2 Related works

Vodrahalli et al. (2020) and Pan et al. (2019) emphasize that clear images are essential in teledermatology for accurate medical diagnoses. Meanwhile, Suha and Sanam (2022) point out that blurry images can easily cause misunderstandings when treating acne, leading to incorrect diagnoses. Straight forward methods such as Wiener filtering and Lucy-Richardson deconvolution (Šroubek et al., 2019; Joshi and Sheetlani, 2019) work well only in stable conditions, as noted by Gnanasambandam et al. (2024). Some works used GANs to fix blurry images because these methods can sharpen photos while keeping them looking natural (Kupyn et al., 2018; Kupyn et al., 2019; Xiong et al., 2024; Peng et al., 2023). Work by Zhai et al. (2022) and Wang et al. (2023) also showed that GANs are even capable of handling the challenge of deblurring facial images. However, keeping small facial details and highlighting contours continues to be a difficult task in face deblurring research (Shi et al., 2024; Qi et al., 2021). Also, works by Lee et al. (2020), Qi et al. (2021), and Jung et al. (2022) have shown that exposing contours remains a challenging task. Meanwhile, Collins et al. (2020) stressed the importance of minor facial feature information for producing the results of improved quality.

Recognizing the importance of clear images in teledermatology and the current challenges in GAN-based facial deblurring, this study focuses on improving these methods for acne imaging through contour accentuation and sketch-based transformations. Martis et al. (2024) and Capozzi et al. (2021) proved that clearly highlighting facial boundaries in training sketches can improve the quality of images produced by sketch-to-photo GANs. Likewise, Mankotia et al. (2024) reported that DeblurGAN performs better when the facial outline is clearly exposed. Building on these findings, Chen et al. (2023) demonstrated that using a preprocessor to expose contours early in the process leads to better deblurring performance. Motivated by this, we incorporated CAT as a preprocessor in this work to achieve better deblurring performance.

## 3 Proposed system

**FIGURE 2:**
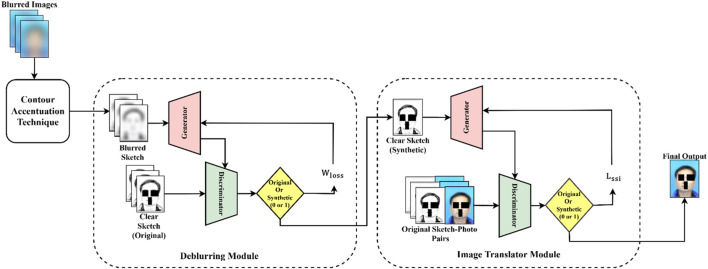
Overview of the proposed image deblurring framework with CAT.

The proposed image deblurring framework consists of three primary components:1) Contour Accentuation Technique (CAT), 2) Deblurring Module, 3) Image Translator module. Figure 2 shows the entire workflow of our system and details of each process are elaborated in subsequent sections.

### 3.1 Contour accentuation technique

**FIGURE 3:**
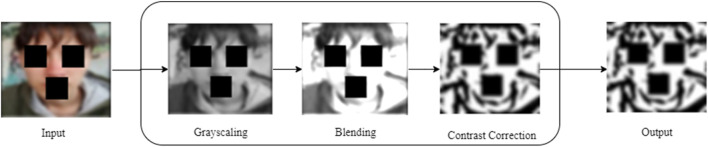
Illustration of proposed CAT process.

This is a preprocessor used to transform the image into a sketch where important features, such as contours, are exposed in blurred image aiding to improve the quality of the image generation further. Figure 3 outlines the steps of CAT, including grayscale, blending, and contrast correction. These steps work together to refine the image, exposing the contours. Each step is discussed in detail in the following subsections.

#### 3.1.1 Grayscale conversion

This step transforms the color image into a grayscale version. This method reduces color information targeting on exposing the facial boundary to reveal the face structure. This step is vital for sketch generation, as it exposes the structural features necessary for creating realistic sketches (Tani and Yamawaki, 2024). Equation 1 reveals the mathematical formulation for the grayscale conversion.

$$Gray\left(x,y\right)=\left[\left(0.299*\;R\left(x,y\right)\right)+\left(0.587\;*\;G\left(x,y\right)\right)+\left(0.114\;*\;B\left(x,y\right)\right)\right]$$ (1)

In the equation, $R\left(x,y\right),G\left(x,y\right),and\;B\left(x,y\right)$ denote the red, green, and blue components of a pixel. The specific values 0.299, 0.587, and 0.114 correspond to the weighting factors used to convert a color image to grayscale in the RGB color space. These values are based on the relative luminance contributions of the red, green, and blue components, respectively, as defined by the ITU-R BT.601 standard. The green component contributes the most to perceived brightness by human eye, which is why it has the highest weighting.

#### 3.1.2 Blending function

In this process, the transitions between light and dark areas in grayscale image is smoothened in order to preserve key details in the facial area (Zhang et al., 2020). Equation 2 explains the blending function used in CAT.

$$B\left(x,y\right)=Gray\left(x,y\right)\;*K\left(x,y\right)$$ (2)

In this equation, $B\left(x,y\right)$ represents the blended image obtained after applying the Gaussian kernel to the original image $I\left(x,y\right)$ through a convolution process. This operation overlaps the kernel on each pixel of the image and computes a weighted average of the surrounding pixels, with weights given by the kernel as per Equation 3.

$$K\left(x,y\right)=\frac{1}{2\pi \sigma^{2}}\times e^{-\frac{x^{2}+y^{2}}{2\sigma^{2}}}$$ (3)

The parameter $\sigma^{2}$ is adjusted to achieve effects, such as enhancing the contrast, sharpening the edges, or smoothing the noise.

#### 3.1.3 Contrast correction

At this stage, blended image is enhanced by adjusting brightness and tuning luminance. For dark images, a luminance value greater than 1 is used, and for light images, less than 1, ensuring realistic and consistent luminance (Chen and Chen, 2022). Additionally, the function adjusts contrast, essential for blending images with different exposures or color temperatures (Dang et al., 2024). Equation 4 demonstrates this process mathematically.

$$V_{out}=\left({\left(\frac{V_{in}}{255}\right)}^{\gamma^{-1}}\right)\times 255$$ (4)

This equation shows how each pixel $V_{in}$ in the blended image is adjusted to produce the output pixel $V_{out}$ after applying the gamma correction. $\gamma^{-1}$ is the inverse of the gamma value, used to lower the original luminance to balance luminance level. The number 255 is used to normalize and denormalize pixel values. Since the system deals with 8-bit gray scale images where pixel values range from 0 to 255, this normalization (divided by 255) scales the pixel values to a range of 0 to 1 , which is necessary for the gamma correction calculation. After the calculation, the pixel values are scaled back (multiplied by 255) to the original range of 0–255.

### 3.2 Deblurring module

The deblurring module takes blurred sketches produced by the CAT as input and outputs deblurred sketches. As shown in Figure 2, this module uses DeblurGAN (Kupyn et al., 2018), trained specifically on facial sketches, to achieve the deblurring process. DeblurGAN enhances blurred sketches by training GAN network that contains generator and discriminator components. The generator consists of a deep convolutional neural network that performs an end-to-end transformation from blurred to deblurred facial sketches. It starts with an initial convolutional layer with 64 filters, a 3×3 kernel size with a step size of one to extract low-level features of the blurred facial sketch, followed by two down-sampling layers with 128 and 256 filters each using a kernel of 3×3 with a step size of 2 to capture its abstract representations. There are nine residual blocks, each filtering 3×3 convolutional layers and skip connections to prevent vanishing gradient problems, facilitating deeper network training. There are two up-sampling layers with 128 and 64 transposed convolution filters for reconstructing the deblurred facial sketch by refining details and reducing the noise factor. Finally, the output layer employs a 7×7 convolutional layer with a Tanh activation function used to normalize the pixel between −1 and 1.

The discriminator module distinguishes between original and synthetic sketches. It employs four convolutional layers with filters 64, 128, 256 and 512 respectively, each using 4 × 4 kernel with step size of 2 (except for the last layer). To enhance training stability and feature extraction, LeakyReLU activation with α=0.2 is utilized. Additionally, batch normalization with momentum value of 0.999 is applied to all layers except the first to further stabilize training. Batch normalization is applied in all layers except the first. The last layer comprises a fully connected layer with one neuron that produces a scalar output to compute the loss that reveals the similarity of clear sketch in correspondence to the original and synthetic sketch. To improve the training process of the model, Wasserstein distance is used (Song and Lam, 2021). Equation 5 describes the mathematical formulation of Wasserstein distance.

$$W_{loss}\left(P_{r},P_{g}\right)=\inf_{\gamma \epsilon \pi \left(P_{r},P_{g}\right)}E_{\left(x,y\right)∼\gamma }\left[\left\|x-y\right\|\right]$$ (5)

In Equation 5, $P_{r}$ and $P_{g}$ ​ represent the original and synthetic probability of pixel intensity distribution of the sketches, respectively, while $\pi \left(P_{r},P_{g}\right)$ denotes the set of all joint distributions γ whose marginals are $P_{r}$ and $P_{g}$ . Additionally, we use the learning rates of 0.0002 for the discriminator with hyperparameters of $\beta_{1}=0.5$ and $\beta_{2}=0.999$ respectively.

### 3.3 Image translator module

The generated sketch from the deblurring module is fed as input to this module. As depicted in Figure 2, thus module uses sketch-to-photo cGAN. Unlike the deblurring module, this module focuses on generating photo-realistic images preserving the structure obtained from the deblurring module. The generator architecture in this case begins with an initial convolutional layer of 64 filters with 4×4 kernel and a step size of two, followed by down sampling layers with 128, 256 and 512 filters using a 4×4 kernel and a step size of two, followed by nine residual blocks featuring 3×3 convolutions and skip connections. The up-sampling layer uses 512, 256, 128 and 64 transposed convolutional filters. Finally, the output layer produces RGB images with a 4×4 kernel with a step size of 1 and Tanh activation function that translates a sketch into a photo-realistic image (Sannidhan et al., 2019).

The discriminator employs a patch-based structure with four convolutional layers with filters 64,128, 256 and 512 respectively using a 4×4 kernel with LeakyReLU activation with α=0.2 and batch normalization with momentum value of 0.999. The last layer produces a scalar output that evaluates adversarial loss. The loss function evaluates the similarity index of the generated photo images against original photos through adversarial and Structural Similarity Index (SSI) values (Osahor et al., 2020; Yu et al., 2019). Equation 6 explains the loss function important for evaluating the similarity between original and generated images.

$$L_{total}=\lambda_{adv}L_{adv}\left(G,D\right)+\lambda_{ssi}L_{ssi}\left(G\right)$$ (6)

In the equation, $L_{total}$ is the total loss function to be minimized by the discriminator. $L_{adv}\left(G,D\right)$ represents the traditional adversarial loss function between the generator G and discriminator D . $L_{ssi}\left(G\right)$ is the loss derived from the Structural Similarity Index (SSI), measuring the similarity between the synthetic images by G and the target original images. $\lambda_{adv}$ and $\lambda_{ssi}$ are weighting coefficients that balance the contribution of adversarial loss and SSI-based similarity loss, respectively. The values chosen for $\lambda_{adv}=0.8$ and $\lambda_{ssi}=0.2$ with hyperparameters of $\beta_{1}=0.5$ and $\beta_{2}=0.999$ respectively.

## 4 Design of the proposed image deblurring framework

This section presents Algorithm 1: Deblurred_Sketch_To_Photo, which details an approach for converting blurred sketches into clear, color photos using GANs integrated with CAT.

Algorithm 1 Deblurred_Sketch_To_Photo.

**Input:** Blurred sketch.
**Output:** Color Photo with improved clarity.
1: Initialize DeblurGAN with generator G and discriminator D

2: **For every blurred image do**
3: $I_{blurred}\leftarrow CAT\left(I_{orginal}\right)$

4: **do**
5: **Generate a deblurred image** $I_{synthetic}=G\left(I_{blurred}\right)$ using G on an image $I_{blurred}$

6: Train D: $I_{synthetic}$ from original $I_{original}$ , to   classify images accurately.
7: **Update G** using **Wasserstein distance** $W\left(P_{r},P_{g}\right)=\underset{\gamma \in \Pi \left(P_{r},P_{g}\right)}{\inf}E_{\left(x,y\right)∼\gamma }\left[\left\|x-y\right\|\right]$

8: Optimize both G and D using their respective   objective functions:
9: For **D, maximize:** $L_{D}=-E\left[D\left(I_{original}\right)\right]+E\left[D\left(I_{synthetic}\right)\right]$ $+\lambda \cdot E\left[{\left(\left|\nabla D\left(I\right){|}_{2}-1\right.\right)}^{2}\right]$

10: For **G, minimize** the **Wasserstein distance** to     adjust its parameters effectively
11: **while** (D fails to distinguish from original     images)
12: **for** the deblurred training sketch samples, **do**
13: **Sample batch** of m **noise samples** from prior     **noise** $p_{g}\left(noise\right)$

14: Sample batch of images from the data
15: update **discriminator** functions $\forall_{\theta d}\left\{\frac{1}{m}\sum_{i=1}^{m}\left[logD\left({p_{x}}^{\left(i\right)}\right)+\log\left(1-D\left(G\left({noise}^{\left(i\right)}\right)\right)\right)\right]\right\}$

16: sample batch of **m noise sample**s for image
17: update the **generator** using gradient     functions. $\forall_{\theta g}\left\{\frac{1}{m}\sum_{i=1}^{m}\log\left(1-D\left(G\left({noise}^{\left(i\right)}\right)\right)\right)\right\}$

18: **end for**
19: **done**

The CAT (Lines 3–5) sharpens blurred images by enhancing edges, converting to grayscale, blending to emphasize structures, and refining pixels for clearer sketches. DeblurGAN (Lines 8–14) employs a GAN framework to enhance sketch clarity, with a generator creating deblurred images and a discriminator refining accuracy by distinguishing them from sharp images. The Sketch_to_Photo_GAN (Lines 17–23) turns deblurred sketches into photorealistic images through iterative training, aligning outputs closely with input sketches.

## 5 Results

This section outlines the outcomes achieved through the experimental investigation of our proposed image deblurring framework. The system was implemented on a high-performance setup with Dual NVIDIA Tesla P100 GPUs, each with 3584 cores and 18.7 Teraflops of power, and Dual Intel Xeon E5-2609V4 CPUs, with 8 cores at 1.7 GHz and 128 GB RAM. To evaluate the outcomes, the following referential metrics are used: SSIM, PSNR, DIQA SRCC, DIQA PLCC, and AE. Additionally, non-referential metrics such as NIQE, BRISQUE, FID, and IS are also employed (Sannidhan et al., 2019; Hu et al., 2023; Martis et al., 2024).

### 5.1 Dataset description

As previously mentioned, this study utilized photographic images from four datasets: 1) the Acne Recognition Dataset (Kucev, 2023), comprising 220 images, 2) the CUHK dataset (Wang and Tang, 2008), containing 188 images, 3) the AR dataset (Cao et al., 2022), featuring 200 images, and 4) the XM2GTS dataset (Messer et al., 1999), which includes 42 images. Using these images required sketches are generated through CAT. These datasets initially contained no blurred images. Therefore, Gaussian blur (Ai et al., 2023) was applied with kernel 5 × 5 to blur the images. The four datasets used in this study were consistently divided into training and testing sets with a split ratio of 70% for training, and 30% each for testing.

### 5.2 Assessment of CAT’s effect on deblurring module

This section explores the impact of CAT on the DeBlurring Module applied to the datasets described in Section 5.1. The effectiveness of CAT in improving image quality is evaluated using the metrics discussed earlier, and the results are presented in Table 1.

**TABLE 1:** Evaluation of CAT’s effectiveness on the deblurring module across datasets.

Evaluation metric	Dataset							
	Acne recognition dataset		CUHK		AR		XM2GTS	
	With CAT	Without CAT	With CAT	Without CAT	With CAT	Without CAT	With CAT	Without CAT
NIQE ↓	2.35	4.01	2.27	3.94	2.55	3.53	2.76	3.35
BRISQUE ↓	28.36	38.15	25.38	37.6	20.1	38.16	38.25	43.25
DIQA SRCC ↑	0.91	0.62	0.97	0.63	0.74	0.72	0.78	0.74
DIQA PLCC ↑	1.01	0.61	0.86	0.56	0.9	0.62	0.85	0.6
SSIM ↑	0.83	0.53	0.86	0.77	0.91	0.54	0.88	0.72
PSNR ↑	22.35	18.21	24.01	21.97	28.48	22.71	30.73	25.37
AE ↓	22.25	94.38	25.35	89.42	38.85	96.15	71.41	122.59
FID ↓	10.77	13.48	10.11	16.23	9.7	13.98	9.81	15.54
IS ↑	3.4	1.86	2.87	1.58	2.92	1.91	3.2	2.08

Table 1 demonstrates that the application of CAT leads to significant improvements across all metrics. Notably, the SSIM shows an average increase of 35% across all datasets. This improvement highlights CAT’s effectiveness in preserving critical structural details.

### 5.3 Assessment of CAT’s effect on the image translator module

This section examines the impact of CAT on the image translator module in producing photo images from sketches. The evaluation utilizes sketches generated by the deblurring module, both with and without CAT, as input to the image translator module. Table 2 summarizes the findings, including both non-referential and referential metrics previously discussed, with the data reflecting average values across all datasets outlined in Section 5.1.

**TABLE 2:** Impact of CAT on image translator module.

Evaluation metric	With CAT	Without CAT
NIQE ↓	2.4	3.6
BRISQUE ↓	28	45
DIQA SRCC ↑	0.89	0.69
DIQA PLCC ↑	0.84	0.65
SSIM ↑	0.85	0.62
PSNR ↑	25	20
AE ↓	40	100
FID↓	15	30
IS↑	3.2	2.5

The data in Table 2 shows significant improvements with CAT across all the metrics confirming the impact of CAT aiding the generation of better-quality photos.

### 5.4 Visual gallery

**FIGURE 4:**
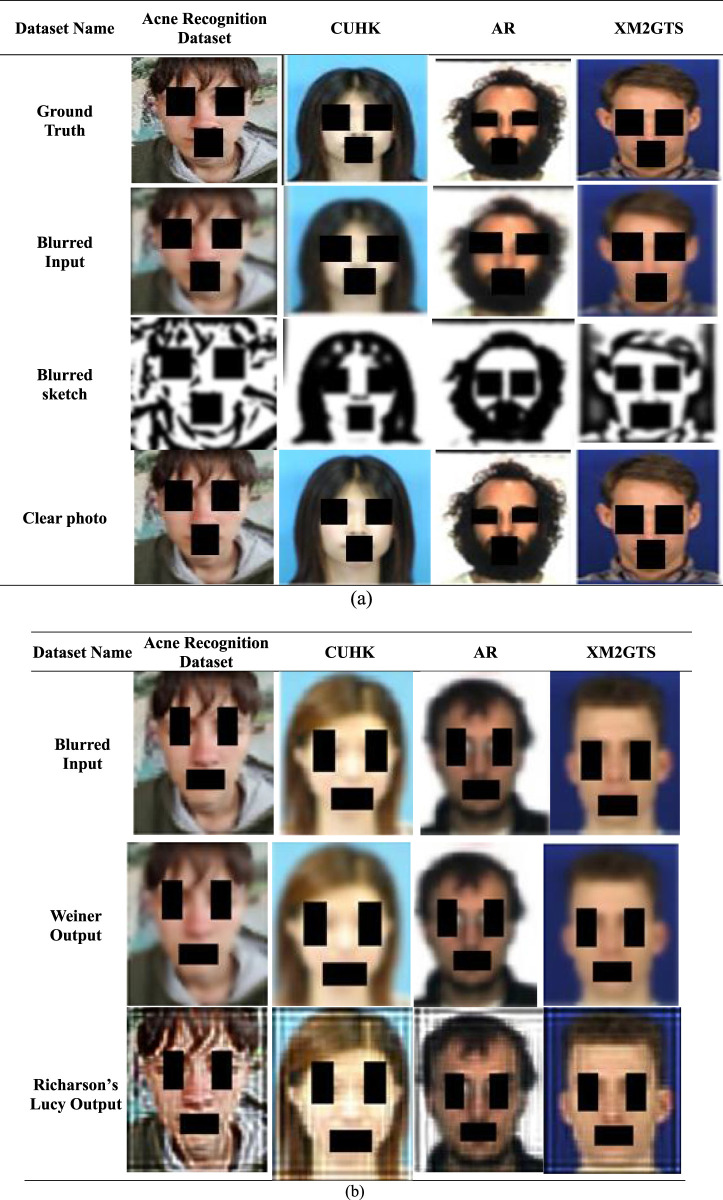
**(A)** Visualization of the proposed image deblurring framework **(B)** Visual comparison of deblurring results using Wiener Filtering and Richardson-Lucy deconvolution.

This section presents a visual gallery depicting image synthesis evolution during training. Figure 4A illustrates the deblurring process across adopted datasets, transitioning from Ground images to Blurred inputs, blurred sketch produced by CAT and clear photo from image translator module. Further 4 b) presents visual analysis of deblurring using Wiener Filtering and Richardson-Lucy Deconvolution.

### 5.5 Comparative analysis with existing approaches

Table 3 presents a comparative analysis of the proposed image deblurring framework against existing image enhancement and GAN-based deblurring techniques. The results represent the average values obtained across the adopted datasets.

**TABLE 3:** Comparison of the proposed image deblurring framework with existing techniques.

Evaluation method	Technique	NIQE ↓	BRISQUE↓	DIQA SRCC ↑	DIQA PLCC ↑	SSIM ↑	PSNR ↑	AE ↓	FID ↓	IS ↑
Comparison of the image deblurring framework against traditional enhancement techniques	Fusion of Median and Wiener filter (Martis and Balasubramani, 2020)	4.2	47	0.53	0.54	0.58	16	135	34	1.05
	Unsharp mask guided filtering (Shi et al., 2021)	3.8	39	0.61	0.67	0.63	18	101	27	1.81
	Adaptive color correction technique (Lin et al., 2023)	3.1	33	0.69	0.71	0.72	20	72	23	2.4
	Proposed image deblurring framework	2.4	28	0.89	0.84	0.85	25	40	15	3.2
Comparison of the image deblurring framework against existing GAN models for deblurring	Semantic face Deblur GAN (Lee et al., 2020)	4.6	80	0.44	0.40	0.49	11	82	49	1.7
	Realistic Deblurring (Lee et al., 2020)	4.1	64	0.49	0.46	0.56	13	78	44	1.9
	Face Image Deblurring (Qi et al., 2021)	3.8	58	0.54	0.57	0.61	16	71	40	2.4
	Guided Face Deblurring (Jung et al., 2022)	3.8	61	0.62	0.69	0.62	18	69	38	2.3
	Style GAN (Collins et al., 2020)	2.7	32	0.65	0.72	0.73	21	65	25	2.4
	ESIDformer (Chen et al., 2023)	3.2	48	0.73	0.76	0.75	14	64	27	2.3
	Proposed image deblurring framework	2.4	28	0.89	0.84	0.85	25	40	15	3.2

## 6 Conclusion

In this paper, we propose an image deblurring framework to address blurring issues in teledermatology, with a particular focus on acne images. The framework is designed to deblur images and enhance their clarity through three core modules: the Contour Accentuation Technique (CAT), the deblurring module, and the image translator module. The CAT module forms the foundation of the framework, extracting facial contours from blurred images and generating a blurred sketch to aid the deblurring process in the next module. The deblurring module enhances the clarity of the sketch produced by the CAT module by removing blur. Finally, the image translator module transforms the refined sketch into a photo image, offering a realistic view. Experimental analysis shows that incorporating CAT significantly improves the quality of the generated photo images. The proposed image deblurring framework on comparison achieved notable results, demonstrating superior performance over existing methods. In future, this research work can be extended to support and automate the acne severity assessment, aiding dermatologists in making faster and more accurate diagnoses.

## Data Availability

The original contributions presented in the study are included in the article/supplementary material, further inquiries can be directed to the corresponding authors.
